# Association between cognitive function and life-space mobility in older adults: results from the FRéLE longitudinal study

**DOI:** 10.1186/s12877-018-0908-y

**Published:** 2018-09-24

**Authors:** François Béland, Dominic Julien, Nathalie Bier, Johanne Desrosiers, Marie-Jeanne Kergoat, Louise Demers

**Affiliations:** 1 Groupe de recherche Solidage, CSSS de la Montagne, site Metro, 1801, boulevard de Maisonneuve Ouest, bureau 600, Montréal, Québec H3H 1J9 Canada; 2 École de santé publique, Département d’administration de la santé, Université de Montréal, C.P. 6128, succ. Centre-Ville, Montréal, Québec H3C 3J7 Canada; 3 Faculté de médecine, Division de médecine gériatrique, Université McGill, Hôpital général juif, 3755, chemin de la Côte-Ste-Catherine, Montréal, Québec H3T 1E2 Canada; 4 Département de psychologie, Université de Montréal, Pavillon Marie-Victorin, C. P. 6128, succ. Centre-Ville, Montréal, Québec H3C 3J7 Canada; 5 Centre de recherche, Institut universitaire en santé mentale de Montréal, 7331, rue Hochelaga, Montréal, Québec H1N 3V2 Canada; 6 Centre de recherche, Institut Universitaire de Gériatrie de Montréal, 4545, Chemin Queen Mary, Montréal, Québec H3W 1W5 Canada; 7 Faculté de médecine, École de réadaptation, Université de Montréal, C.P. 6128, succ. Centre-Ville, Montréal, Québec H3C 3J7 Canada; 8 École de réadaptation, Faculté de médecine et des sciences de la santé, Université de Sherbrooke, 3001, 12e Avenue Nord, Sherbrooke, Québec J1H 5N4 Canada; 9 Centre de recherche sur le vieillissement, Centre intégré universitaire de santé et de services sociaux de l’Estrie, Centre hospitalier universitaire de Sherbrooke, 1036, rue Belvédère Sud, Sherbrooke, Québec J1H 4C4 Canada; 10 Faculté de médecine, Département de médecine, Université de Montréal, C.P. 6128, succ. Centre-Ville, Montréal, Québec H3C 3J7 Canada

**Keywords:** Cognition, Life-space, Mediation, Moderation, Aging

## Abstract

**Background:**

Cross-sectional and longitudinal studies show conflicting results regarding the association between cognition and life-space mobility, and little is known regarding the mediators and moderators of the association.

The aim of this study was to investigate the association between cognition and life-space mobility in older adults, as well as the intervening variables modifying the relationship.

**Methods:**

Community-dwelling older adults aged 65 years and older (*N* = 1643) were assessed at three time points over a period of 2 years. Growth mixture models with mediation and moderation analysis were utilised to investigate association between cognitive function and life-space mobility. The potential mediators and moderators were depressive symptoms, locus of control, gait speed and grip strength. Analysis was controlled for age, sex, education, annual income, number of chronic illnesses, and living site.

**Results:**

The direct association between initial scores of cognitive function and life-space was mediated by initial scores of depressive symptoms and gait speed, and moderated by initial scores of grip strength. No direct association between change in cognitive function and change in life-space mobility was found; the scores were mediated by change in depressive symptoms.

**Conclusions:**

We conclude that the relationship between change in cognitive function and life-space mobility in older adults is not well-defined over an observation period of 2 years.

**Electronic supplementary material:**

The online version of this article (10.1186/s12877-018-0908-y) contains supplementary material, which is available to authorized users.

## Background

The concept of life-space mobility is receiving growing attention in the field of aging. It defines a spectrum of geographic areas that extend from domicile to distant destinations [1]. Constricted life-space mobility has been associated with adverse health outcome, including illness, poor self-rated health, difficulty in basic and instrumental activities of daily living, [1] depressive symptoms, [1, 2] frailty, and death [2].

Webber and colleagues have presented a comprehensive framework illustrating variables associated with life-space mobility [3] (Webber framework). Five categories of determinants of life-space mobility were hypothesized: (i) cognitive determinants are relevant for reaching distant destinations; (ii) psychosocial determinants affects the motivation to be mobile; (iii) physical performance reflects the influence of strength and balance; (iv) environmental context reflects the influence of the built environment; and (v) financial resources, which influences activities, access to mobility aids and modes of transportation. According to the Webber framework, all categories of determinants are influenced by age, culture, and personal life history.

The association between cognition and life-space mobility has been investigated in the literature, but the direction of the relationship remains unsettled. Although mobility may stimulate cognition, high levels of cognitive function may be a prerequisite for being mobile. Mobility is a complex activity requiring high levels of physical and cognitive function, especially when venturing beyond one’s domicile [4]. A decline in higher-order cognitive abilities might be followed by a decline in the more complex aspects of life-space mobility, such as driving [5]. Older adults can also adjust their mobility behavior in a way that reflects their cognitive skills [6].

Empirically, cross-sectional studies have reported significant association between higher cognition and an expansive life-space, [2, 7–10] although discrepant findings were reported [11–13]. To our knowledge, three longitudinal studies have investigated whether baseline cognitive function was associated with future life-space mobility, all with mixed results [14–16].

Determinants may influence each other and interact to shape mobility, as suggested in the Webber framework. This opens the possibility that intervening variables mediate or moderate [17] the association between cognition and life-space mobility. Studies have shown that psychosocial determinants (depression or locus of control) and physical determinants (grip strength or walking speed) are related to cognition [9, 18–20] and to life-space mobility [1, 2, 8, 9, 11]. These determinants are legitimate, intervening variables in change in both cognition and mobility. However, with few exceptions, [9] the role of psychosocial and physical determinants as mediators or moderators in the association between cognition and life-space mobility has not been investigated.

Using the Webber framework, the current study examines the association between cognition and life-space mobility in community-dwelling older adults. It also focusses on the role of depressive symptoms and locus of control as psychosocial determinants, and the role of gait speed and grip strength as physical determinants. Both determinants are considered to be proximate forces modifying the association of change in cognition and physical mobility. Other determinants in the Webber framework are considered control variables. Specifically, the main research questions are:

1. Is change in cognition associated with change in physical mobility?
2. Do depression, locus of control, gait speed, and grip strength intervene in the association of change between cognition and mobility? If so,
  - Do they play intervening roles along the continuum of values of the measurement instruments, from the lower to the higher end?
  - Or, is there a threshold at which they start to intervene?

**Fig. 1:**
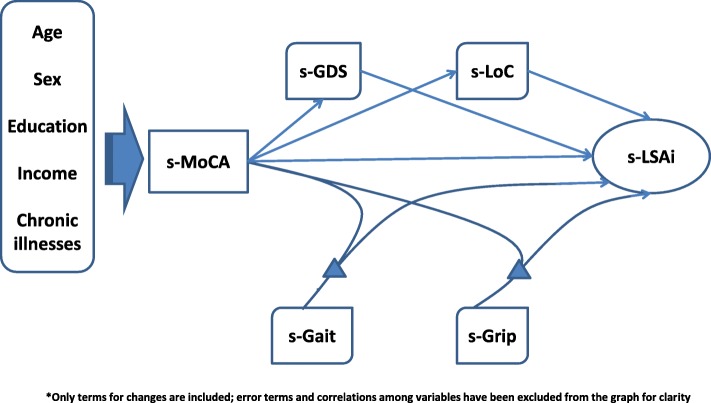
Theoretical Model^*^

The first question pertains to the main issue raised within the Webber framework. The second question identifies four proximate intervening variables, while questions 2.a and 2.b specify the mechanisms – mediation and moderation - through which these variables act. Figure 1 models the relationship among the six variables, including control variables. Mediation is illustrated by using psychological determinants, and moderation by using the physical determinants.These research questions were examined using data from a longitudinal study of frailty – FRéLE (*Fragilité: étude longitudinale de ses expressions* [21]). Growth mixture models were used, simultaneously testing the association among the set of variables at baseline, or at a fixed state, and the association over time, or the rate of change [22]

.

## Methods

### Participants and design

The FRéLE study sampled community-dwelling older adults (aged 65 and over) from three locations in the province of Québec, Canada: a metropolitan area (Montréal); a mid-sized city (Sherbrooke); and a small town and surroundings (Victoriaville). Participants were recruited in 2010 by a random sample drawn from the Québec Medicare database. The sample was stratified by sex, age, and location to secure a sufficient number of participants presenting with frailty. Individuals with significant hearing problems, or inability to speak either French or English were excluded. From the database, 4915 individuals were identified and 432 were excluded based on the study screening criteria. Of the 4483 qualified individuals, 52.2% refused to participate. Of the remaining 2141 individuals, 20% dropped out of the survey before T0 (baseline), 2% could not be contacted, and 1.3% did not complete the questionnaire, leaving a total of 1643 participants in the study (see Additional file 1: Part 1 for sample characteristics and Additional file 5).

Participants underwent a series of functional and psychosocial measurements carried out by trained health professionals at baseline (T0), and two annual follow-ups (T1, T2). When moderate to severe cognitive issues were suspected, the consent of a trustee or fiduciary was obtained. The research protocol was approved by the Research Ethics Committee of the Jewish General Hospital (#15–182). Preliminary analysis revealed that the sociodemographic characteristics and health status of FRéLE participants reflected the community-dwelling elderly population across the province [23].

### Measures

#### Predictor variable

##### *Cognitive function* (MoCA)

Cognitive function was measured using the Montreal Cognitive Assessment instrument [24]. MoCA scores ranged from 0 to 30, with lower scores representing greater cognitive impairment. The test-retest reliability of the MoCA is high, along with its internal consistency [24]. FRéLE respondents with scores above 23 on the Mini-Mental State Evaluation [25] were given the MoCA. The 66 respondents excluded from taking the MoCA were considered to have a lower cognitive status. They were not excluded from the analysis and were censured to the left. In the current study, continuous MoCA scores were used.

#### Predicted variable

##### *Life-space mobility* (LSA)

The Life-Space Assessment index [1] was used to investigate life-space mobility. In the LSA, five different types of life-space are assessed: within-home, around home, neighborhood, town and outside of town. For each type of life-space, participants reported the frequency of movement during the previous 4 weeks, and whether assistance was needed. LSA scores ranged from 0 to 120, with higher scores reflecting greater life-space mobility. The LSA shows excellent test-retest reliability over a two-week period [1].

#### Intervening variables

##### *Depressive symptoms* (GDS)

Depressive symptoms were assessed using the 15-item version of the *Geriatric Depression Scale* [26]. GDS scores ranged from 0 to 15, with higher scores indicating higher levels of depressive symptoms. In order to align GDS scores with other time-varying variables scores, they were transformed: high scores indicated lower depressive symptoms. Continuous GDS scores were used in this study.

##### *Locus of control* (LoC)

The Personal Mastery Scale [27] is a seven-item scale assessing the extent of the belief that one is able to control or influence outcomes. For each item, scores ranged from 1 (strongly agree) to 4 (strongly disagree), with higher scores reflecting a higher level of control.

##### *Gait speed* (gait)

Walking speed (cm/sec) was assessed by recording the time required to walk 4, 3, or 2.44 m (according to the space available in the participant’s home) [28].

##### *Grip strength* (grip)

Handgrip strength was measured using the hand Baseline vigorimeter, following American Society of Hand Therapists recommendations [29]. Three measures for each hand were taken, alternating between the dominant and non-dominant hand. For the current study, the variable represented the average score in KiloPascal (KPa) of the three measures from the strongest hand.

#### Control variables

Control variables were assessed by a series of items on sex, age, education, annual income, number of chronic illnesses, and living site (metropolitan area, mid-sized city, small town) [30].

### Statistical analysis

#### Modelling change on time-varying variables

The predictor variable (MoCA), the predicted variable (LSA), and the four intervening variables (Gait, Grip, GDS, and LoC) are time-varying indicators, as is one of the control variables (number of chronic illnesses). Change in each of the seven variables over the three observed time periods was modelled using growth modelling [31] with non-ignorable dropout cases [32]. At each period, the average of the time-varying variables at T0 was subtracted from the corresponding score. LSA, MoCA, gait speed, and grip strength scales were transformed by dividing each by a constant in order to align the range of variance values among all variables [22].

**Fig. 2:**
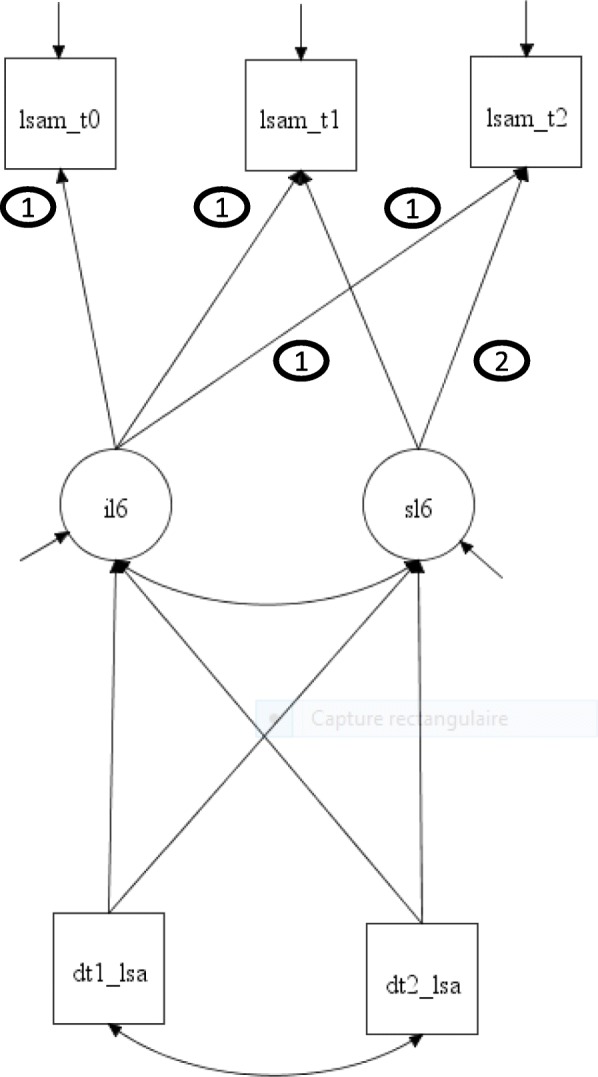
Growth model: One variable

Observed scores for LSA (Lsam_t0; Lsam_t1; Lsam_t2) at the three time periods are shown in Fig. 2 with rectangles; above the rectangles, linked with arrows, are the associated standard errors (not shown). Over three time periods, the variables are patterned by two parameters: their intercepts (*iL6-*type), representing state or cross-sectional results; and the slope (*sL6-*typ*e*), representing change (increase, decrease, or stability). Coefficients for intercept with observed scores at each period are fixed to “1”, and for slope to “0”, “1” and “2” [31]. Change parameters for T0 are fixed to zero for identification purposes. Inasmuch as non-null standard errors are associated with intercept, respondent scores vary. Though slope may signal changes, the standard errors may not be significantly different from zero, inasmuch as the amount of change is not different among respondents. In this case, the cohort of respondents changes collectively in the same way from one time period to another.The first step in the analysis estimates the growth model parameters for each predicted and predictor variable and for the intervening time-varying variables, adjusting for control variables. Level of significance was set at α ≤ 0.05. The set of non-statistically significant growth model coefficients is then fixed to zero and tested to examine whether they can be excluded from further analysis using bootstrap likelihood ratio test (BLRT) and Bayesian information criterion (BIC).

Pattern-mixture modelling (PMM) is used to model non-ignorable dropout cases (Fig. 2) [32, 33]. PMM does not explore causal effects of observed variables on dropout [32]. Rather, sources of variation in the outcome variables are identified.

#### Modelling change in LSA and MoCA

The association of MoCA with LSA, adjusting for control variables, is estimated using three terms: the regression of the intercept of LSA (*iLSA*) on 1) the intercept of MoCA (*iMoCA*); and the regression of the slope of LSA (*sLSA*) on 2) *iMoCA* and 3) *sMoCA*.

Parameter estimates for MoCA and for LSA growth models (Table 1) may change with the regression of LSA on MoCA. For example, the coefficient for dropout may lose statistical significance, inasmuch as MoCA and LSA contribute to loss of respondents between panels.

**Table 1:** Parameter estimates for growth models

		Bootstrap CI			Bootstrap CI		
		Coef.	CI<0,95	CI>0,95	Coef.	CI<0,95	CI>0,95
A. Predictor and predicted variables							
		LSA/10			MoCA/2		
Fixed							
Average (i)		0.42	-0.03	0.87	0.02	-0.65	0.72
Growth rate (s)		-0.28*	-0.52	-0.08	0.57***	0.34	0.83
Random							
Average (i)		2.55*	2.23	2.93	4.57***	3.78	5.54
Growth rate (s)		0.21*	0.06	0.32	0.38**	0.07	0.64
(i) x (s)		-0.23*	-0.42	-0.04	0.16	-0.16	0.55
Pattern-Mixture: Missing not at random							
(i) Time_1		0.07	-0.29	0.43	-0.30	-0.76	0.12
(i) Time_2		-0.42**	-0.70	-0.11	-0.63**	-1.07	-0.23
(s) Time_1		-0.15	-0.37	0.01	-0.11	-0.40	0.12
(s) Time_2		-0.15	-0.37	0.01	-0.11	-0.40	0.12
Observed variables residual variances							
@t0		1.58*	1.21	1.97	1.08*	0.59	1.64
@t1		1.33*	1.14	1.56	1.36*	0.93	1.77
@t2		1.11*	0.77	1.43	1.41*	0.69	2.31
		LL	# of par.	BIC	LL	# of par.	BIC
Log-likehood	Model: H1^1^	-16244.00	36	32751	-16778.00	36	33824
	Model: H0^2^	-16245.20	34	32738	-16779.50	34	33850
	-2(LL[H1]-LL[H0]	2.40	2	13	3.00	2	-26
B. Intermediate variables							
		Gait speed/10			Grip Strenght/10		
Fixed							
Average (i)		0.84*	0.35	1.31	-0.13	-0.45	0.20
Growth rate (s)		-0.12	-0.31	0.08	-0.14*	-0.26	-0.03
Random							
Average (i)		4.49*	4.04	5.14	1.99*	1.82	2.24
Growth rate (s)		0.18	-0.03	0.40	0.02	-0.06	0.10
( i) x (s)		-0.23	-0.51	0.02	-0.01	-0.09	0.08
Pattern-Mixture: Missing not at random							
(i) Time_1		0.01	-0.38	0.36	-0.05	-0.32	0.20
(i) Time_2		-0.64*	-0.94	-0.33	-0.09	-0.29	0.14
(s) Time_1		-0.13	-0.32	0.06	-0.16*	-0.29	-0.02
(s) Time_2		-0.13	-0.32	0.06	-0.16*	-0.29	-0.02
Observed variables residual variances							
@t0		2.37*	1.94	2.89	0.92*	0.76	1.01
@t1		1.63*	1.39	1.90	0.40*	0.32	0.49
@t2		1.02*	0.62	1.47	0.56*	0.41	0.73
		LL	# of par.	BIC	LL	# of par.	BIC
Log-likehood	Model: H1^1^	-16841.60	36	33950	-14848.00	36	29963
	Model: H0^2^	-16844.20	31	33918	-14852.60	30	29927
	-2(LL[H1]-LL[H0]	5.20	5	32	9.20	6	36
		GDS			Locus of control		
Fixed							
Average (i)		0.00	-0.48	0.46	-0.24	-0.78	0.37
Growth rate (s)		-0.33*	-0.57	-0.13	0.06	-0.21	0.35
Random							
Average (i)		4.06*	3.62	4.70	6.06*	5.32	7.03
Growth rate (s)		0.54*	0.33	0.76	0.87*	0.59	1.23
(i) x (s)		-0.32*	-0.58	-0.07	-0.78*	-1.22	-0.43
Pattern-Mixture: Missing not at random							
(i) Time_1		0.19	-0.19	0.53	-0.25	-0.69	0.19
(i) Time_2		0.64*	0.34	0.92	-0.40	-0.71	0.01
(s) Time_1		0.05	-0.14	0.24*	0.04	-0.27	0.29
(s) Time_2		0.05	-0.14	0.24	0.04	-0.27	0.29
Observed variables residual variances							
@t0		1.32*	0.85	1.80	2.80*	2.12	3.50
@t1		1.87*	1.64	2.14	3.82*	3.48	4.29
@t2		0.74*	0.32	1.17	2.45*	1.80	3.16
		LL	# of par.	BIC	LL	# of par.	BIC
Log-likehood	2003Model: H1^1^	-16797.80	36	33862	-18108.40	36	36484
	Model: H0^2^	-16798.70	33	33841	-18109.30	32	36455
	-2(LL[H1]-LL[H0]	1.80	3	21	1.80	4	29

* *p* ≤ 0,05; ** *p* ≤ 0,01; *** *p* ≤ 0,001

^1^H1: Models with all coefficients estimated

^2^H0: Models with non-significant coefficients fixed to 0

^3^LSA and Grip Strength scores were divided by 10; MoCA by 2

Muthén and Muthén [22] proposed the decomposition of complex models into parts, integrating the parts using resulting estimated parameters as starting values. This strategy was used in building the model to test our main hypotheses (Fig. 1). As in Cheong et al., [34] shapes of the growth trajectory for each intervening variable were investigated.

#### Mediating and moderating the association of MoCA with LSA

**Fig. 3:**
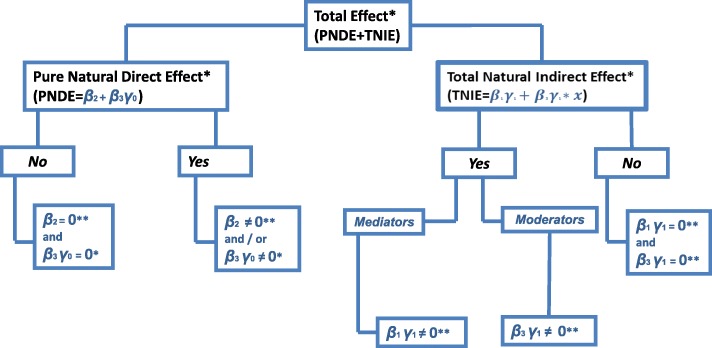
Direct effects, mediators and moderators: A web of hypotheses

The direct association of MoCA with LSA, and the mediation and moderation of psychosocial characteristics and physical performance between MoCA and LSA are estimated with the Muthén and Asparouhov [31] web of hypotheses found in Fig. 3. The terms in Fig. 3 were derived from the expected value of LSA conditional on MoCA, and on the intervening variables [31]. Thus, the expected value of LSA is conditional based on:

1. The coefficient of MoCA: β_2_. This is the Baron and Kenny [17] estimation of the direct effect of MoCA on LSA;
2. The product of the coefficient of a mediator and MoCA with the coefficient of LSA and this mediator: β_1_ϒ_1_. This is the Baron and Kenny [17] formulation of the mediation hypothesis, with extensions by MacKinnon et al. [35] and Preacher et al.; [36]
3. The term for moderators: β_3_ϒ_1_. This is homologous with the Hayes index [37] of moderated mediation when the interaction involves the predictor and a mediator variable. In the Baron and Kenny scheme, [17] the interaction term β_3_ alone defines moderation; [38]
4. The term β_3_ϒ_0_. Because ϒ_0_ is not involved in the association of MoCA with intervening variables, [31] β_3_ϒ_0_ does not contribute to indirect association, but to direct association. Thus, even though β_2_ = 0, the direct association of MoCA with LSA can be significant.

Modeling intercept and slope introduces restrictions among the associations. Slope represents change occurring across all observation points, while intercept involves observations at baseline only. Thus, slope cannot predict intercept, but intercept can predict slope. The Parallel Process Latent Growth model [34] is an example. This model has been extended to include: [39] 1) the intercept-only model, where intercepts are sole mediators; 2) the slope-only model, where slopes are sole mediators; and 3) the multiple mediation model, where both intercepts and slopes are mediators. Intercepts are not mediators for other intercepts in any of these models. Thus, in these models, the structure of association among the intercepts is considered ignorable [40]. The FRéLE design is observational and longitudinal. Two data generating processes are proposed as expressions of the theoretical model of change originating from the Webber framework: 1) observations at baseline resulting from the cumulative operation of the model over the life-course, and 2) changes during the longitudinal observational period (2 years). This two-way process requires that the mediation and moderation structure is imposed on both intercepts and slopes.

Null hypotheses, as shown in Fig. 3, were tested using the Mplus constraint procedure [22]. Standard errors in the indicators for mediator and moderator were bootstrapped for all models, except for the model with two interaction terms. The bootstrap procedure could not be applied in this latter case, as the computer time required to obtain estimates became unwieldy on a 2-Xeon-based machine with 32 threads.

## Results

At baseline, the average score on the LSA original scale was 62.6, decreasing somewhat over time. The MoCA score was below the threshold of 26 for normal cognitive functioning, [24] but increasing from baseline to T1. Grip strength was the only intervening variable with a continuing, decreasing trend. Gait speed was slightly above the cut-off point for acceptable functioning (8 cm/sec), with an average of 8.8 cm/sec. Gait speed and both psychosocial determinants were stable.

### Estimating change in time-varying variables

Preliminary analysis was conducted to ensure that change observed in time-varying variables could not be confounded by unstable, unreliable measurements. Results suggested that change could not be attributed to measurement errors (Additional file 2: Part 2).

Table 1 provides the parameter estimates of modelling change for each of the intervening variables, adjusted for control variables. There were significant variations in respondent scores. Growth rates were negative on LSA, gait speed, grip strength, and GDS, and positive on MoCA. Dropouts had lower scores than respondents who remained in the study at T1, suggesting that the estimated positive values of growth rate on MoCA may be a survival effect. Though growth rate for LoC did not change significantly between periods, variations in respondent growth rates were obtained. Finally, except for MoCA, residual errors were smaller at T2 than at T0, indicative of a decrease in the heterogeneity of respondents (Additional file 3: Part 3).

### Regression of MoCA and LSA

The coefficients for the regression of MoCA and LSA, with control variables included in the model, are shown in Table 2. The growth rates for MoCA and LSA and their random terms were more or less in the same range as estimates in Table 1. However, the association of *iMoCA* with *sMoCA* lost statistical significance. Only the regression coefficients of the LSA intercept (*iLSA*) on MoCA intercept (i*MoCA*) were significant. Thus, if life-space increased with MoCA, a change in life-space was not sensitive to levels and change in MoCA.

**Table 2:** Regression of LSA on MoCA^a^

		Bootstrap CI		
		Coef.	CI < 0,95	CI > 0,95
Fixed				
LSA	Intercept (iLSA)	0.356^*^	0.031	0.684
	Growth rate (sLSA)	−0.284^***^	−0.432	−0.107
MoCA	Intercept (iMoCA)	0.086	−0.612	0.777
	Growth rate (sMoCA)	0.484^***^	0.359	0.619
Pattern-Mixture: Missing not at random				
LSA	(i) Time_1	0.000	[NS]	[NS]
	(i) Time_2	−0.294^**^	−0.522	− 0.073
	(s) Time_1	0.000	[NS]	[NS]
	(s) Time_2	0.000	[NS]	[NS]
MoCA	(i) Time_1	0.000	[NS]	[NS]
	(i) Time_2	−0.807^***^	−1.149	−0.499
	(s) Time_1	0.000	[NS]	[NS]
	(s) Time_2	0.000	[NS]	[NS]
Random				
LSA	Intercept (iLSA)	2.507^***^	2.180	2.860
	Growth rate (sLSA)	0.271^***^	0.114	0.426
	(iLSA) x (sLSA)	−0.297^**^	−0.477	−0.099
MoCA	Intercept (iMoCA)	4.633^***^	3.906	6.604
	Growth rate (sMoCA)	0.397^*^	0.125	0.642
	(iMoCA) x (sMoCA)	0.128	−0.196	0.474
Regression: LSA				
iLSA on	iMoCA	0.168^***^	0.123	0.217
sLSA on	iMoCA	− 0.002	−0.012	0.028
sLSA on	sMoCA	0.040	−0.231	0.216

^*^p ≤ 0,05; ^**^ *p* ≤ 0,01; ^***^ *p* ≤ 0,001

^a^Control variables included

### Estimating interactions

Following the Muthén and Muthén procedure, [22] interactions among the intervening variables and MoCA were grouped in six parts, one for each intervening variable intercept and slope with significant standard error (Table 3). Interaction was defined by the rule that *iLSA* cannot be regressed on slope terms for MoCA and intervening variables, and intervening variable intercept terms cannot be regressed on s*MoCA*. Intervening variable intercept terms were accepted as potential moderators between *sMoCA* and *sLSA* inasmuch as their association with *sMoCA* was not statistically significant. Latent interaction terms were obtained with the “xwith” operator in Mplus, [22] as in Luo et al. [41]. No interaction terms involving GDS and LoC were significant (Table 3, Part 2). The interaction of gait speed (*iGait*), and grip strength (*iGrip*) with *iMoCA* was significant (Table 3, Part 1) Interaction of *iGrip* with *sMoCA* almost reached significance for *sLSA* at the 0.05 level. Significant interaction terms, plus interaction of *iGrip* with *sMoCA* for *sLSA*, were introduced in a model including control variables, and the predictor, intervening, and predicted variables (Table 4). Interaction terms for *iLSA* were statistically significant when tested one at a time, but their BIC statistics were nearly equal to BIC for the model without interaction (Table 4, Part A.1). Also the null hypothesis for the interaction term for *sLSA* could not be rejected (Table 4, Part A.1). Both interaction terms for *iLSA* were entered in a model (Table 4, Part B.1), and the contribution of each examined. The contribution of *iGait* was not significant, while the null hypothesis for the interaction term with *iGrip* was rejected (Table 4, Part B.2). Finally, the significance of the interaction involving *iGrip* was tested against a new null model including the term for *iGrip*. The null hypothesis of no interaction was rejected (Table 4, Part B.3) and the interaction term of *iMoCA* with *iGrip* for *iLSA* was the only interaction retained in the final model.

**Table 3:** Interactions of intermediate variables with cognitive impairments

	Coef.	s.e.	*P*-level	Coef.	s.e.	*P*-level
Part 1: Physical performance	Gait speed [iGait]			Grip strength [iGrip]		
iLSA1 on interaction of iMoCA2 with:	−0,019**	0.007	0.008	−0,027*	0.011	0.014
sLSA1 on interaction of iMoCA2 with:	0.003	0.004	0.452	0.001	0.006	0.905
sLSA1 on Interaction of sMoCA2 with:	−0.029	0.019	0.121	−0.049	0.026	0.057
	-2LL	# of free Parameters	BIC	-2LL	# of free parameters	BIC
With interaction: [LLh1]	−33,550.4	66	67,589	−31,759.1	67	64,014
Without interaction: [LLh0]	−33,556.3	63	67,579	−31,764.9	64	64,004
−2*(LLh0-LLh1)	11,7*	3	−10	11,7**	3	−10
Part 2: Pyschosocial GDS [iGDS] GDS [sGDS]	GDS [iGDS]			GDS [sGDS]		
iLSA1 on interaction of iMoCA2 with:	0.005	0.008	0.514	N.A.**	N.A.**	N.A.**
sLSA1 on interaction of iMoCA2 with:	−0.037	0.061	0.542	0.006	0.023	0.803
sLSA1 on Interaction of sMoCA2 with:	0.165	0.260	0.526	0.187	0.380	0.623
	-2LL	# of free parameters	BIC	-2LL	# of free parameters	BIC
With interaction: [LLh1]	−33,586.5	71	67,699	−33,585.9	70	676
Without interaction: [LLh0]	−33,587.2	68	67,678	−33,585.6	68	67,678
−2*(LLh0-LLh1)	1.5	3	−21	−0.6	2	67,002
	Locus of control [iLoC]			Locus of control [sLoC]		
iLSA1 on interaction of iMoCA2 with:	−0.006	0.006	0.321	N.A.**	N.A.**	N.A.**
sLSA1 on interaction of iMoCA2 with:	< 0,001	0.003	0.914	−0.01	0.012	0.369
sLSA1 on Interaction of sMoCA2 with:	−0.033	0.027	0.222	−0.052	0.088	0.557
	-2LL	# of free parameters	BIC	-2LL	# of free parameters	BIC
With interaction: [LLh1]	−35,006.1	70	70,530	−35,007.3	69	70,525
Without interaction: [LLh0]	−35,008,0	67	70,512	−35,008,0	67	70,512
−2*(LLh0-LLh1)	3.7	3	−18	1.4	2	−13

*p≤ 0,05

**Not available

**Table 4:** Testing interactions of Cognitive Impairments with Gait Speed and Grip Strength in the final model

	# of free			Gait speed [iGait^3^]			Grip strength [iGrip^4^]		
	-2LL	Parameters	BIC	Coef.	s.e.	*P*-level	Coef.	s.e.	*P*-level
Part A.1: Testing interactions one at a time:									
Without interaction: [LLh4]	−58,867.3	115	118,586						
iLSA^1^ on iGrip + Int[iGrip]^5:^ [LLh5]	−58,858.9	117	118,584				−0.033^***^	0.010	0.001
−2*(LLh4-LLh5)	16.84^***^	2	-2						
iLSA^1^ on iGait + Int[iGait]^6:^ [LLh6]	−58,862.6	116	118,584	−0.018^*^	0.007	0.014			
−2*(LLh4-LLh6)	9.46^**^	1	-2						
sLSA^1^ on iGrip + Int[sGrip]^7:^ [LLh7]	−58,865.0	117	118,596				−0.051	0.031	0.095
−2^†^(LLh4-LLh7)	4.48	2	10						
Part B.1: Testing simultaneously, for iLSA, Int[iGrip] & Int[iGait]									
iLSA on iGrip + Int[iGrip] + Int[iGait]: [LLh8]	− 58,858.7	118	118,591	−0.011	0.008	0.141	−0.026^*^	0.011	0.020
−2^†^(LLh4-LLh8)	17.09^***^	3	5						
Part B.2: Testing the significance of Int[iGrip] and Int[iGait]									
Excluding Int[iGait]: [LLLh9]	−58,858.9	117	118,584						
−2^†^(LLh9-LLh8)	0.25	1	−7						
Excluding Int[iGrip]: [LLh10]	−58,862.6	116	118,584						
−2^†^(LLh10-LLh8)	7.63^*^	2	7						
Part B.3: Testing the significance of Int[iGrip]									
Without interaction + [iGrip]: [LLh11]	−58,865.7	116	118,590						
−2^†^(LLh11-LLh5)	13.69^***^	1	-6						

^*^p ≤ 0,05; ^**^ *p* ≤ 0,01; ^***^ *p* ≤ 0,001

^†^Interactions are not defined for changes in moderators with the LSA intercept

(1) iLSA: intercept for LSA; sLSA: Slope for LSA

(2) iMoCA: intercept for MoCA; sMoCA: Slope for MoCA

(3) iGait: intercept for Gait speed

(4) iGrip: intercept for Grip strenght

### Introducing intervening variables

**Fig. 4:**
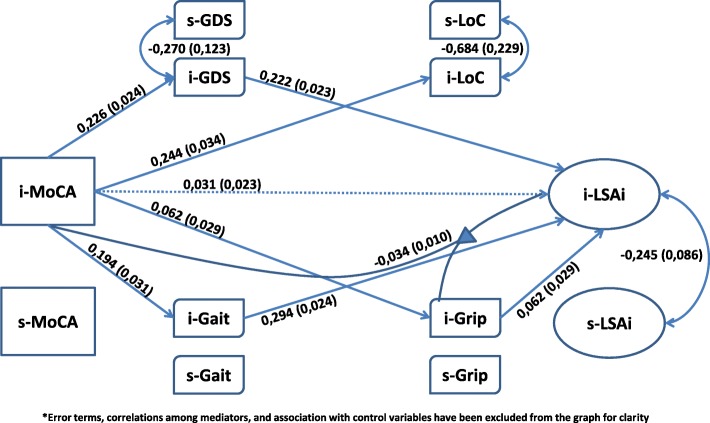
Mediators and Moderators: MoCA’s intercept & LSA’s intercept^*^

**Fig. 5:**
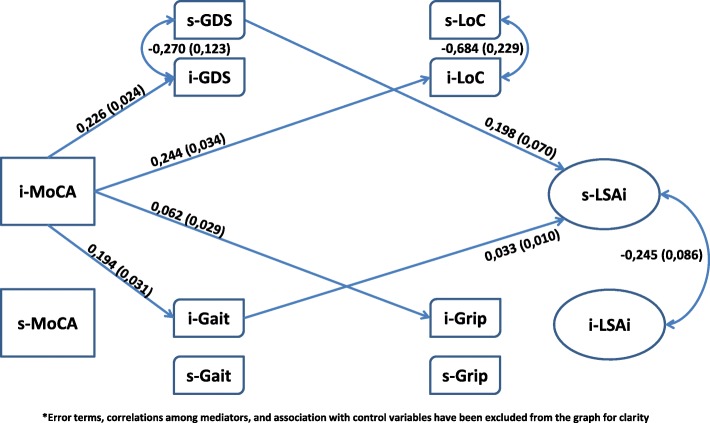
Mediators and moderators: MoCA’s intercept & LSA’s slope^*^

**Fig. 6:**
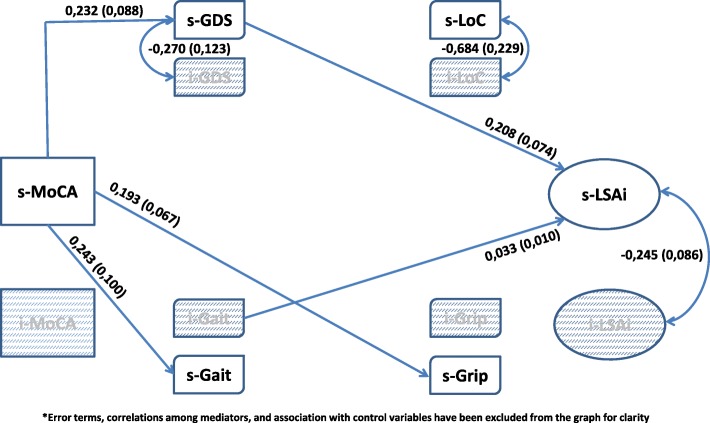
Mediators and moderators: MoCA’s slope & LSA’s slope^*^

A single model was set up that simultaneously included all statistically significant parameters from the above analytical steps. Parameters were excluded if both the estimates and the associated log likelihood ratio tests did not reach statistical significance. Results are shown in Figs. 4, 5 and 6. These figures are organized based on MoCA and LSA intercept and slope terms for clarity. Also, only the significant structural parameters involving association among intercept and slope for *MoCA*, the intervening variables, and LSA are shown. Control variables and residuals have been excluded from these figures for greater clarity.

The model for the association of *iMoCA* with *iLSA* is shown is Fig. 4. Though the direct association between the two is not significant, it was included in the model because of the *iMoCA-iGrip* interaction terms. Thus, the possibility that *iGrip* is a moderator was examined. All intervening variables were shown to be associated with *iMoCA* and all, except *iLoC*, were associated with *iLSA. iGDS*, *iGait* and *iGrip* were tested as mediators. Estimated regression parameters involving *sLSA* and *iMoCA* are shown in Fig. 5. *sLSA* was associated with *iGait* and *sGDS*. Only *iGait* may have a mediator relationship with *iMoCA* and *sLSA*. Finally, in Fig. 6, the terms for change are emphasized to enhance the association among *sMoCA* and *sLSA,* and the intervening variables. No direct association was found between the two variables. Also, *sMoCA* is associated with *sGait*, *sGrip* and *sGDS*. As the *sGait* and *sGrip* standard errors were fixed to zero, no association with sLSA could be estimated. In this case, only *sGDS* could be considered to be a mediator. In summary, the set of our main hypothesized mediations, except for LoC, was potentially retained for mediation involving intercept only. *iGrip* was retained as a moderator between i*MoCA* and *iLSA*. The term for change on LSA (*sLSA*) was not related to the intercept or slope for MoCA. *sGDS* and *iGait* were associated with *sLSA*, and *sGDS* almost reached statistical significance as a mediator between *sMoCA* and *sLSA*.

### Statistical significance of mediator and moderator terms

The term for direct effect (β_3_ϒ_0_) was not statistically significant. All mediator and moderator relations shown in Figs. 4, 5 and 6 were statistically significant, except for the mediation of *iGrip* between *iMoCA* to *iLSA*. However, *iGrip* remained a statistically significant moderator (table not shown).

**Fig. 7:**
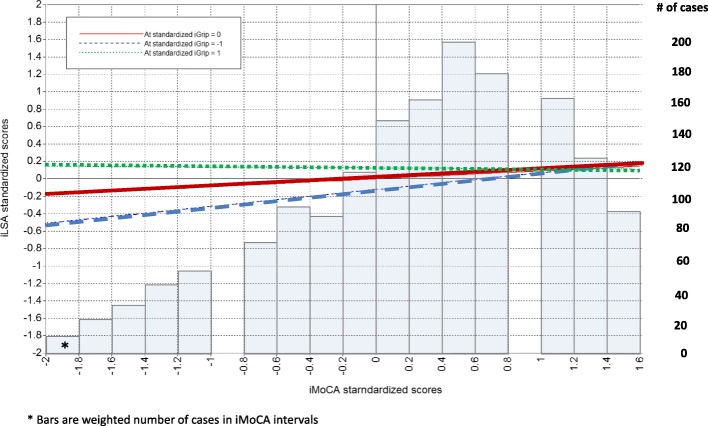
Averages standardized iLSA scores predicted by iMoCA at three levels of standardized scores for iGrip

Figure 7 shows three regression lines for standardized *iLSA* scores on standardized *iMoCA* scores. The association of *iMoCA* with *iLSA* is progressively headed toward zero as *iGrip* scores increase. *iGrip* appears to compensate for the association of *iMoCA* with *iLSA* at lower scores on *iMoCA*. Thus, the association of *iMoCA* with *iLSA* is significant only for low *iGrip* scores. However, statistical significance is not a measure of association. Statistically significant coefficients in this study were small (Additional file 4: Part 4).

The weighted distribution of the number of FRéLE respondents on the *iMoCA* continuum is shown in Fig. 7 as shaded bars. The modification of the association of *iMoCA* with *iLSA* at low levels of *iGrip* concerns a minority of respondents.

## Discussion

The aim of this study was to examine the longitudinal association between cognitive decline and change in life-space mobility in community-dwelling older adults, and to investigate the impact of moderators and mediators on this association. Based on the Webber framework, two questions were asked. The first examined the extent that change in cognitive function was associated with change in life-space mobility. In a model with cognition as the sole predictor, including controlled variables, the direct association was not significant. To our knowledge, no other studies have examined associations between change in cognitive function and change in life-space mobility. In our study, the direct association between the two variables was significant at baseline, suggesting an accumulation of reciprocal effects throughout the life course. Yet, with the introduction of intermediate psychosocial and physical performance variables, this direct association fell into the non-statistically significant range. Some studies did not report cross-sectional associations between cognition and life-space mobility, [11–13] while others showed a relationship [2, 7–10]. Discrepancies between these findings may be explained by heterogeneity in samples (e.g., Canadians, Americans female, Mexican Americans), sample size (ranging from 300 to 2737), age of respondents (e.g., 65 years and older, 75 years and older, 65–74 years), and country in which the studies were conducted (USA, Canada, Brazil, Columbia, Finland). That is, associations between cognition and life-space mobility may be influenced by individual as well as sociocultural factors.

The second question dealt with the mediation and mediator roles of psychosocial variables (depression and Locus of Control) and physical performance variables (gait speed and grip strength), and their potential to affect the association between change in cognitive function and life-space mobility. Our results showed a weak mediating effect for change in depression. Locus of control was not associated with change in life-space mobility; it had no mediating nor moderating roles. In contrast, one study reported that locus of control moderated association between cognition and life-space mobility at baseline [9]. Baseline values on gait speed were related with change in life-space mobility. Though change was observed at the cohort level in gait speed and grip strength, the change rate did not vary among individuals. The association of change in gait speed and grip strength with change in life-space mobility could not be estimated.

At baseline, gait speed played a mediating role in the association between cognition and life-space mobility. Examined separately, interactions of gait speed and grip strength with cognition were statistically significant. However, only the interaction of grip strength remained statistically significant when both were entered simultaneously in the structural equation. Grip strength played a moderating role at baseline only: the association of cognition with life-space mobility decreased with increasing grip strength up to a point where the association had no statistical significance. Of the psychosocial variables, baseline depression showed a trend towards mediating association between cognitive function and life-space mobility. Locus of control was not associated with life-space mobility. Our results, at baseline, thus replicate those of studies reporting an association between higher depressive symptoms and lower life-space mobility [1, 2, 8].

Changes were observed in this study over a two-year period, a short period of time by any standard. Nonetheless, change in cognition and life-space mobility has been observed. Locus of control did not change at the population level, but change in individuals was observed. Grip strength decreased chronologically at the same level across the entire FRéLE sample.

To sum up our results, in persons aged 65 and over, change in life-space mobility occurs slowly and is not related to cognition at the population level. Only change in depression and gait speed at baseline was associated with change in life-space mobility. A low level of association was obtained between the Webber framework determinants and life-space mobility.

Our results support the fact that slow change, observed over time throughout the aging process is complex and not well-understood. Our results also suggest that the process of change in life-space mobility extends over a long period of time, including the entire life course. Thus, from a population health perspective, persons aged 65 and over cumulate life-long social, psychosocial, and health experiences, and any change in health status, from then on, depends on their life course. Whether or not conclusions can be drawn on the efficacy of population health interventions on slowly changing issues observed at a population level is a difficult topic. Whether public health interventions or policies on physical performance at the population level could affect the process of change in life-space mobility, given changes in cognitive ability, is still an unanswered question within the framework of this paper. Mobility in itself is a determinant of health [42]. Indeed, mobility is essential to create and maintain relationships and to participate socially [43]. Social participation has been associated with benefits to physical, cognitive and mental health of seniors [44]. Therefore, future studies should focus on a better understanding of the impact of determinants of health on mobility as well as the impact of mobility on health in seniors, in light of the Webber framework.

There are some limitations to the current study. First, although longitudinal, the study was not designed to determine the direction of causal processes in cognitive deficit, life space, psychosocial characteristics, or physical function, but rather to estimate the source of variations of change in life-space mobility within the Webber framework. Our research questions were legitimate, given our use of growth curve modelling and our understanding of causality in an observation-based longitudinal study design. Causality was not examined, but we tested and used null hypotheses for parameters derived from a structural equation model to get insight on the absence of causality in the set of predictor, intermediate, and predicted variables.

Second, association and interaction among the intermediate variables have not been considered. Also, it could be argued that some of the controlled variables should have been considered for moderation effects. Modeling these associations and interactions would have increased the already complex structural models tested in this paper, pushing to the limit our ability to obtain reliable parameter estimates.

Third, FRéLE was not designed to be representative of the older population in the three locations. However, the distribution of FRéLE characteristics does reflect the elderly population across Quebec.

Fourth, respondents lost to follow-up had lower scores on predictor, intermediate, and predicted variables, than those who remained in the study. However, these sources of variations attributable to non-ignorable missing cases were included in models. Fifth, only changes occurring in a short period of time were observed in the FRéLE study. However, significant changes in cognition and life-space, and psychosocial variables were observed over a two-year period. The variables in this study may require an observation period of longer than 2 years for their association in change to appear. We have not been able to identify latent classes for change in life-space mobility, suggesting that patterns of change are uniform in FRéLE respondents, whatever their age. It is possible that latent classes for change in the predictor and intermediate variables exist. However, estimating latent classes for change on multiple variables introduces complexity in the modelling of the structural equations, in the interpretation of results, and in the estimation procedures. Future work in this direction is under consideration (Additional file 5).

## Conclusions

In conclusion, differences in respondents at baseline may have resulted from sequences of multiple processes over their life course: resilience, recovery, improvement or sustained decline [45]. The baseline variables of association, mediation and moderation could be considered as a starting point to examine life course processes. The type of processes that individuals, population subgroups, and whole populations experience could have resulted in different population health states, at baseline and over the course of our study. The FRéLE study was not set up to identify these processes. Nonetheless, locating FRéLE baseline results within a life course perspective opens up opportunities for new studies. In a life course perspective [46] where the baseline model (Fig. 4) can be considered the result of cumulative processes, a strong case can be made in retaining a mediating hypothesis for physical performance variables and depression, and a moderating hypothesis for grip strength. Though the *iGait* interaction term was not significant when it was considered with *iGrip*, difficulties in estimating multiple interaction terms in structural equation models suggest caution before rejecting the moderation hypothesis for *iGait* in future studies. Finally, correlated change in cognitive process and life-space at the population level seems to occur at a low level over all ages, 65 and older. A two-year period may be too short to capture these changes. Also, the association of cognition with life-space mobility seems to be limited to a small proportion of people, as suggested by the moderator role of grip strength. In effect, association of cognition with mobility was limited to lower scores on grip strength, while the sample grip strength scores were skewed toward middle and high scores. Finally, baseline results can be considered a cross-sectional sample, while growth-modeling results are derived from a longitudinal design. In any case, the cross-sectional data and longitudinal data in the FRéLE sample did not yield the same results.

## Additional files

Additional file 1: Part 1 Characteristics of respondents. Describes the characteristics of the FRéLE sample. (DOCX 21 kb)

Additional file 2: Part 2 Reliability of the time-varying variables. Examines the reliability of the time-varying variables to ensure that change observed in these variables was not confounded by unstable reliability of the measures. (DOCX 28 kb)

Additional file 3: Part 3 Estimating change in each of the time-varying variables. Describes change in each of the time-varying variables. (DOCX 25 kb)

Additional file 4: Part 4 Statistical significance and strength of association. Describes change in the predicted variable based on a 1-unit change in the predictor variable. (DOCX 23 kb)

Additional file 5: **Table S1.** Characteristics of FRéLE participants. Table presenting descriptives of the FRéLE sample. (DOCX 26 kb)

## Abbreviations

BIC: Bayesian information criterion
BLRT: Bootstrap likelihood ratio test
FRéLE: Fragilité: étude longitudinale de ses expressions [Frailty: A longitudinal study of its expression]
Gait: Gait speed
GDS: Geriatric depression scale
Grip: Grip strength
*i*: Intercept
KPa: KiloPascal
LoC: Locus of control
LSA: Life-space assessment
MOCA: Montreal cognitive assessment
PMM: Pattern-mixture modelling
*s*: Slope
