# Supplementary material for: Association between cognitive function and life-space mobility in older adults: results from the FRéLE longitudinal study
Source: BMC Geriatr. 2018 Sep 24;18:227. doi: 10.1186/s12877-018-0908-y (PMC6154880; doi:10.1186/s12877-018-0908-y)
Supplement: Supplementary file 1 — Part 1 Characteristics of respondents. Describes the characteristics of the FRéLE sample. (DOCX 21 kb) [file 12877_2018_908_MOESM1_ESM.docx]

**Supplemental material**

*Part 1. Characteristics of respondents*

The FRéLE sample included as many men as women and the observed average age was 78 (Supplemental Table 1). The number of respondents was the same for each of the three sites. Non-weighted average income and education were respectively C$28,235 and 10.7 years. Both were significantly higher in the metropolitan area (C$33,851; 12.6 years), followed by the mid-sized city (C$26,352; 10.5 years), and the small town (C$23,543 and 8.9 years). The number of chronic diseases was significantly higher in the mid-sized city (3.6), and lower in both the metropolitan area and small town (3.1).
