# Supplementary material for: Association between cognitive function and life-space mobility in older adults: results from the FRéLE longitudinal study
Source: BMC Geriatr. 2018 Sep 24;18:227. doi: 10.1186/s12877-018-0908-y (PMC6154880; doi:10.1186/s12877-018-0908-y)
Supplement: Supplementary file 2 — Part 2 Reliability of the time-varying variables. Examines the reliability of the time-varying variables to ensure that change observed in these variables was not confounded by unstable reliability of the measures. (DOCX 28 kb) [file 12877_2018_908_MOESM2_ESM.docx]

**Supplemental material**

*Part 2. Reliability of the time-varying variables*

Reliability for MoCA, GDS, and LoC was investigated to ensure that change observed in these variables was not confounded by unstable reliability. LSA has a complex scoring algorithm. The reliability study [[1](#_ENREF_1)] for LSA uses a test-retest design, which was unavailable here. However, the Baker et al. [[1](#_ENREF_1)] set of validating measures was used to examine stability over time of the association of LSA with physical performance (SPPB [[2](#_ENREF_2)]), SF36 physical and mental components, [[3](#_ENREF_3)] GDS, [[4](#_ENREF_4)] comorbid conditions, [[5](#_ENREF_5)] self-reported health disability, and a questionnaire based on activities of daily living (ADL [[6](#_ENREF_6)]) and instrumental activities of daily living (IADL [[7](#_ENREF_7)]). Stability of reliability coefficients for gait speed and grip strength was not assessed; both were measured using standardized performance tests.

Reliability coefficients varied throughout the panels within a range of 0.02 for MoCA (T0: 0.78; T1: 0.80; T2: 0.80), 0.03 for GDS (T0: 0.75; T1: 0.78; T2: 0.78), and 0.04 for LoC (T0: 0.70; T1: 0.73; T2: 0.74). Ranges of variation in correlation of LSA with the Baker et al. [[1](#_ENREF_1)] validating measurements set between panels, were at a low of 0.02 and a high of 0.04 for correlation varying in absolute value from ±0.30 to ±0.60. Estimated correlation in FRéLE was in the same range as correlation obtained by Baker et al.^1^ except for BADL (FRéLE: -0.60; Baker et al. [[1](#_ENREF_1)]: -0.39) and for comorbid conditions (FRéLE: -0.30; Baker et al. [[1](#_ENREF_1)]: -0.19). Our exploration into the variability of reliability and of validating measures concludes that changes in MoCA, GDS, LoC, and LSA were likely not attributable to measurement errors. Evidence of the stability of reliability was not available for grip strength or gait speed, however interviewers were systematically trained and retrained before and during data collection periods to ensure consistent procedure was followed.

**References**

1. Baker PS, Bodner EV, Allman RM: **Measuring life‐space mobility in community‐dwelling older adults**. *Journal of the American Geriatrics Society* 2003, **51**(11):1610-1614.

2. Guralnik JM, Simonsick EM, Ferrucci L, Glynn RJ, Berkman LF, Blazer DG, Scherr PA, Wallace RB: **A short physical performance battery assessing lower extremity function: association with self-reported disability and prediction of mortality and nursing home admission**. *Journal of gerontology* 1994, **49**(2):M85-M94.

3. Ware Jr JE, Sherbourne CD: **The MOS 36-item short-form health survey (SF-36): I. Conceptual framework and item selection**. *Medical care* 1992:473-483.

4. Yesavage JA, Sheikh JI: **9/Geriatric depression scale (GDS) recent evidence and development of a shorter version**. *Clinical gerontologist* 1986, **5**(1-2):165-173.

5. Groll DL, To T, Bombardier C, Wright JG: **The development of a comorbidity index with physical function as the outcome**. *Journal of clinical epidemiology* 2005, **58**(6):595-602.

6. Katz S, Ford AB, Moskowitz RW, Jackson BA, Jaffe MW: **Studies of illness in the aged: the index of ADL: a standardized measure of biological and psychosocial function**. *Jama* 1963, **185**(12):914-919.

7. Lawton M, Brody E: **Assessment of older people: self-maintaining and instrumental activities of daily living**. *The Gerontologist* 1969, **9**(3):179-186.
