# Supplementary material for: Association between cognitive function and life-space mobility in older adults: results from the FRéLE longitudinal study
Source: BMC Geriatr. 2018 Sep 24;18:227. doi: 10.1186/s12877-018-0908-y (PMC6154880; doi:10.1186/s12877-018-0908-y)
Supplement: Supplementary file 3 — Part 3 Estimating change in each of the time-varying variables. Describes change in each of the time-varying variables. (DOCX 25 kb) [file 12877_2018_908_MOESM3_ESM.docx]

**Supplemental material**

*Part 3. Estimating change in each of the time-varying variables*

Scores on LSA decreased between time periods at the rate of -0.284 (Table 1 of the main text). Confidence intervals and levels of significance were obtained using the Mplus bootstrap procedure. [[1](#_ENREF_1)] The cumulative decreased from T0 to T2 reaching -0.853 (CI_0,95:_ -1.568; -0,240), on a variable with a 12 point range. Rate of change among respondents varied significantly (see Table 1 CI_0,95_ in the main text). Also, dropouts had lower scores at T1 on LSA than remaining respondents at T2. The association of intercept with growth rate was significant and negative, indicative of decreasing change with lower intercepts. Finally, heterogeneity of respondent scores on LSA decreased from T0 to T2 as residual errors decreased.

The growth rate for MoCA was positive and statistically significant, increasing from T0 to T2 (CI_0,95:_1.070; 2.480) in a range of 0-15. The dropout scores at T1 were lower than for those who remained in the study by a factor of -0.630. The growth rate random coefficient was statistically significant. Also, residual error increased between T0 and T2, indicative of increasing heterogeneity over time.

All indicators of physical function changed in an expected, negative direction, but only the growth rate for grip strength is significant. The random term associated with growth rate was not statistically significant, and yielded parallel changes in respondents. [[2](#_ENREF_2)] Thus, growth rate was the same for all respondents from T0 to T2. Though growth rate for gait speed was not significant, dropouts had lower rates for gait speed at T1 than those who remained in the study at T2. Also, both of these indicators of physical function are more homogeneous at T2 than at T0, based on residual error.

Only GDS had a change in growth rate that was negative and significant among the indicators of psychosocial characteristics. The LoC coefficient was positive, small and not statistically significant. Dropouts had lower depression scores at T1 than those who remained in the study. Heterogeneity on GDS and LoC was lower at T2 than at T0.

Models with non-statistically significant coefficients fixed at zero (the null models: H0) were tested and compared to models shown in Table 1 in the main text (non-null models: H1), using BLRT statistics and BIC adjustment criteria. Using BLRT, H0 models were not rejected, and BIC criteria for H0 models are lower than the BIC criteria for H1 models. Thus, H0 models fit the data well. Parameter estimates for H0 models were used as starting values in subsequent tests.

**References**

1. Muthén LK, Muthén BO: **Mplus User’s Guide**, Seventh Edition edn. Los Angeles, CA: Muthén & Muthén; 1998-2017.

2. Cheong J, MacKinnon DP, Khoo ST: **Investigation of mediational processes using parallel process latent growth curve modeling**. *Structural Equation Modeling* 2003, **10**(2):238-262.
