# Supplementary material for: Association between cognitive function and life-space mobility in older adults: results from the FRéLE longitudinal study
Source: BMC Geriatr. 2018 Sep 24;18:227. doi: 10.1186/s12877-018-0908-y (PMC6154880; doi:10.1186/s12877-018-0908-y)
Supplement: Supplementary file 4 — Part 4 Statistical significance and strength of association. Describes change in the predicted variable based on a 1-unit change in the predictor variable. (DOCX 23 kb) [file 12877_2018_908_MOESM4_ESM.docx]

**Supplemental material**

*Part 4. Statistical significance and strength of association*

To what extent did change in life-space mobility (*sLSA*: mean range over 1-year ±2SD: -4.3 – 3.3) vary with change in cognition (*sMoCA*: mean range over 1-year ±2SD: -5.5 – 6.1)) and changes in depression (*sGDS*)? An increase (decrease) of 1 point over a one year period in MoCA resulted in an increase (decrease) of 0.281 points (0.95CI: -0.019;0.501) to just slightly under zero in *sLSA*. Also, to what extent are two FRéLE respondents different from one another at baseline on life-space mobility (*iLSA*) given their status on cognition (*iMoCA*) and on mediating variables (*iGDS*, *iGait*, *iGrip*)? With *iMoCA* as the sole predictor of i*LSA* and added control variables, an average difference among respondents with 1.00 point on the original *iMoCA* scale (range ±2SD: 17 – 30) resulted in a difference of 0.82 points among respondents on the original *iLSA* scale (range ±2SD: 13 – 112). When all paths from *iMoCA* to *iLSA* were included, as shown in Figure 4 (main text), with the interaction term at zero the resulting average difference was 1.40 points (0.95CI: 0.95;1.85) on the original LSA scale and a 1.00 point difference on the original MoCA scale, with the mediation role of *iGDS*, *iGait* and *iGrip*. The interaction with *iGrip* modifies this pattern, with further decreasing scores on *iLSA* and lower scores in *iMoCA* (Figure 7, main text).
