# Supplementary material for: Association between cognitive function and life-space mobility in older adults: results from the FRéLE longitudinal study
Source: BMC Geriatr. 2018 Sep 24;18:227. doi: 10.1186/s12877-018-0908-y (PMC6154880; doi:10.1186/s12877-018-0908-y)
Supplement: Supplementary file 5 — Table S1. Characteristics of FRéLE participants. Table presenting descriptives of the FRéLE sample. (DOCX 26 kb) [file 12877_2018_908_MOESM5_ESM.docx]

**Supplemental material**

Supplemental Table 1. Characteristics of FRéLE participants

| Variables |  | T0 (baseline) | |  | T1 | |  | T2 | |
| --- | --- | --- | --- | --- | --- | --- | --- | --- | --- |
|  |  | % or means | N or s.d. |  | % or means | N or s.d. |  | % or means | N or s.d. |
| Control variables | |  |  |  |  |  |  |  |  |
| Age | 6-74 | 32.6% | 536 |  | -- | -- |  | -- | -- |
|  | 75-84 | 33.8% | 555 |  | -- | -- |  | -- | -- |
|  | 85+ | 33.6% | 552 |  | -- | -- |  | -- | -- |
| Gender | Female | 49.8% | 818 |  | -- | -- |  | -- | -- |
|  | Male | 50.2% | 825 |  | -- | -- |  | -- | -- |
| Education | Years | 10.7 |  |  | -- | -- |  | -- | -- |
| Income | CAD$ | 28235 |  |  | -- | -- |  | -- | -- |
| Location | Metropolitain | 34.0% | 559 |  | -- | -- |  | -- | -- |
|  | Urban | 33.0% | 542 |  | -- | -- |  | -- | -- |
|  | Small town | 33.0% | 542 |  | -- | -- |  | -- | -- |
|  |  |  |  |  |  |  |  |  |  |
| Predicted and predictor variables | |  |  |  |  |  |  |  |  |
| MoCA | range: 0-30 | 23.89 | 3.99 |  | 24.48 | 4.04 |  | 24.34 | 4.03 |
| Life Space index | range: 0-120 | 62.6 | 24.9 |  | 60.6 | 24.1 |  | 61.1 | 24.5 |
|  |  |  |  |  |  |  |  |  |  |
| Intervening variables | |  |  |  |  |  |  |  |  |
| Grip strength | Unit: kiloPascal | 59.2 | 22.6 |  | 55.6 | 21.1 |  | 53.5 | 21.8 |
| Gait speed | Unit: cm/sec | 8.8 | 3.1 |  | 8.6 | 2.8 |  | 8.6 | 3.0 |
| Depressive symptoms | Range: 0-15 | 2.9 | 2.8 |  | 2.8 | 2.6 |  | 2.8 | 2.7 |
| Locus of Control | Range 2-26 | 20.5 | 3.1 |  | 20.4 | 3.1 |  | 20.46 | 3.2 |
